# Advances in Control Strategies against *Spodoptera frugiperda*. A Review

**DOI:** 10.3390/molecules26185587

**Published:** 2021-09-15

**Authors:** Francisco A. Paredes-Sánchez, Gildardo Rivera, Virgilio Bocanegra-García, Hadassa Y. Martínez-Padrón, Martín Berrones-Morales, Nohemí Niño-García, Verónica Herrera-Mayorga

**Affiliations:** 1 Unidad Académica Multidisciplinaria Mante, Universidad Autónoma de Tamaulipas, El Mante 89840, Tamaulipas, Mexico; faparedes@docentes.uat.edu.mx (F.A.P.-S.); berrones.martin@gmail.com (M.B.-M.); nngarcia@docentes.uat.edu.mx (N.N.-G.); 2 Centro de Biotecnología Genómica, Instituto Politécnico Nacional, Reynosa 88710, Tamaulipas, Mexico; gildardors@hotmail.com (G.R.); vbocanegra@ipn.mx (V.B.-G.); 3 Subdirección de Enseñanza e Investigación, Hospital Regional de Alta Especialidad de Ciudad Victoria “Bicentenario 2010”, Cd. Victoria 87087, Tamaulipas, Mexico; hadassayufo@gmail.com

**Keywords:** *Spodoptera frugiperda*, biological control, chemical control, extracts, metabolites

## Abstract

The strategies for controlling the insect pest *Spodoptera frugiperda* have been developing over the past four decades; however, the insecticide resistance and the remarkable adaptability of this insect have hindered its success. This review first analyzes the different chemical compounds currently available and the most promising options to control *S. frugiperda*. Then, we analyze the metabolites obtained from plant extracts with antifeedant, repellent, insecticide, or ovicide effects that could be environmentally friendly options for developing botanical *S. frugiperda* insecticides. Subsequently, we analyze the biological control based on the use of bacteria, viruses, fungi, and parasitoids against this pest. Finally, the use of sex pheromones to monitor this pest is analyzed. The advances reviewed could provide a wide panorama to guide the search for new pesticidal strategies but focused on environmental sustainability against *S. frugiperda*.

## 1. Introduction

The fall armyworm, *Spodoptera frugiperda* (J.E. Smith, 1797), is a lepidopteran insect of the family Noctuidae. The larval stage of this pest has a food preference for leaves and tender shoots, especially buds, becoming a chewer of plant tissue [1]. Its feeding habits make it a polyphagous, migrating, destructive pest of crops in the Western Hemisphere. It also has a high capacity for dispersal and adaptation and a preference for a variety of host plants [2,3].

Because of its behavior in the field, *S. frugiperda* is considered a constant pest in the Americas and recently has also invaded the crops of Africa, India, and China. *Spodoptera frugiperda* pests are present almost all year, causing damage to food crops and as a result economic loss. This situation provokes the misuse use of chemical insecticides (as type of insecticide used, increases in recommended doses of application, number of applications per season/year, and time and rate of application), generating undesirable effects on the environment and humans, and has led to the development of *S. frugiperda* resistance [4,5]. In addition, susceptibility to control methods depends on the growth stage/size of *S. frugiperda*, presenting a greater susceptibility in the early growth stages (first instar) to the different control strategies (*Bacillus thuringiensis* (Berliner, 1915), spinosad, pyrethroid, carbamate and organophosphate insecticides) [6,7].

Another feature of this pest, which has possibly given it fame among crop pests, is the process of divergence, that is, the crossing of biotypes. Such behavior was detected in 1986 by Pashley and co-workers through studies on feeding behaviors and allozymes by PCR techniques and sequence level [8,9,10].

Two biotype strains of *S. frugiperda*, known as “rice” and “corn,” have been identified based on the genetic diversity in the *COI* mitochondrial gene, determined by the fact that organisms belonging to the biotype corn have a high preference for crops of corn, sorghum, and cotton crops. In contrast, the rice biotype prefers rice and grass [9,11], is sensitive to the type of host plant, and presents a differential behavior to the control strategies, the corn biotype being more resistant to *Bacillus thuringiensis* and chemical insecticides (carbaryl, diazinon, cypermethrin, methyl parathion, and methomyl) than the rice biotype [9,12]. All these characteristics led researchers to regard it as a plague of global economic importance.

In this work, we review the use of chemical compounds, plant extracts, and metabolites derived from plants, organisms, and sex pheromones implemented or proposed as new strategies for controlling *S. frugiperda*, updating the effective doses used, structure–activity relationship of different molecules, susceptibility in the biological cycle, involved biological receptors, and new reports of distribution of natural enemies of *S. frugiperda*.

## 2. Chemical Insecticides

Chemical insecticides have been used since 1940 as the most common weapon for pest control in plants because they are the most effective, offering relatively quick and easy application and satisfactory results. Despite some disadvantages, modern agriculture can hardly maintain high yields without chemical input [13]. Most of these problems have resulted from insecticide and pesticide misuse and overuse. In the case of *S. frugiperda*, its eating behavior causes the larvae to be “protected” by the inner leaves of the plant, usually covered with their excrement, making interaction with the insecticide or pesticide difficult. Therefore, farmers must apply the pesticide in the early days of planting, almost directly on the ground or by granulates applied directly to the plant bud. The constant misuse of chemical compounds in these control strategies has generated *S. frugiperda* resistance. Carvalho et al. [14] reported in Brazil *S. frugiperda* strains with 18- and 28-fold resistance to organophosphate and pyrethroid insecticides compared with susceptible strains. This situation has allowed the insect to increase its population density, causing insecticide resistance to compounds such as DDT, cyclodiene organophosphates, carbamates, and pyrethroids, generating the need for new molecules with potential activity [15,16,17].

**Figure 1:**
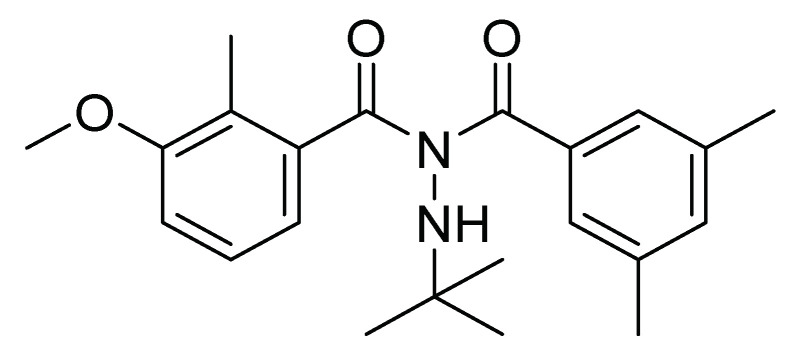
Chemical structure of methoxyfenozide.

In this regard, methoxyfenozide (*N*′-tert-butyl-*N*′-(3,5-dimethylbenzoyl)-3-methoxy-2-methylbenzohydrazide) was developed. Methoxyfenozide is a molting accelerating compound (MAC). Its chemical structure (Figure 1) mimics the biological function of the hormone 20-hydroxyecdysone, which induces premature molt and death due to direct stimulation of ecdysteroid receptors. Methoxyfenozide is a diacylhydrazine compound characterized by lepidopteran insecticidal activity. It acts on the third-instar larvae of *S. frugiperda*, mainly by ingestion, since by the topic application presents a partial action in addition to a low ovicidal action [18,19].

**Figure 2:**
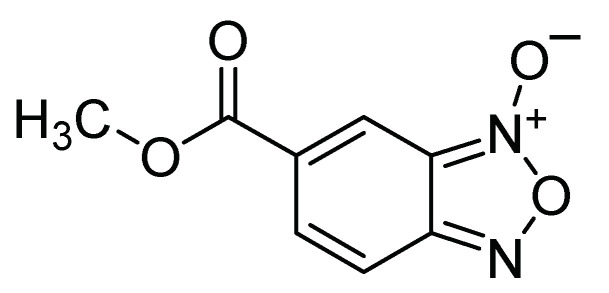
Chemical structure of QX5 (benzofuroxan methyl-5-carboxylate N-oxide).

Another group of chemical insecticides is quinoxaline derivatives. The presence of the quinoxaline family has been described in natural products such as peptides, vitamins, and pharmaceuticals, and they have shown low to moderate toxicity in humans [20,21,22]. In the agricultural area, quinoxaline derivatives and di-N-oxides have been reported as active ingredients in pesticides and herbicides, with mechanisms of action on receptors such as phosphodiesterase in orders such as Blattodea and on cholinesterase in Coleoptera, Diptera, Hemiptera, and Lepidoptera insects. Considering the above, Rosas-García et al. [23] evaluated, by topical bioassays and by ingestion, five compounds derived from N-oxide, on the first instar of three Mexican populations of *S. frugiperda*. All three populations were sensitive to compound QX5 (benzofuroxane methyl-5-carboxylate *N*-oxide) (Figure 2), with 100% mortality in the ingestion bioassay. It is important to mention that some derivatives of 1,4-di-*N*-quinoxaline dioxides obtained through classical synthesis methods have been reported with cytogenetic, mutagenic, and genotoxic effects. However, currently, ecological methodologies have been developed to synthesize quinoxalines with recyclable catalysts, and every day green organic synthesis is gaining ground in the agricultural area, allowing the synthesis of quinoxaline derivatives, which have been found in species such as *Curcuma longa* L.; quinoxaline polymers have also been obtained by green chemistry protocols, which have demonstrated environmental stability. Therefore, it can be indicated that the use of organic synthesis methodologies that respect the environment and with the help of computational molecular docking tools may allow the bioactivity and selectivity of these molecules to be enhanced [24,25,26].

**Figure 3:**
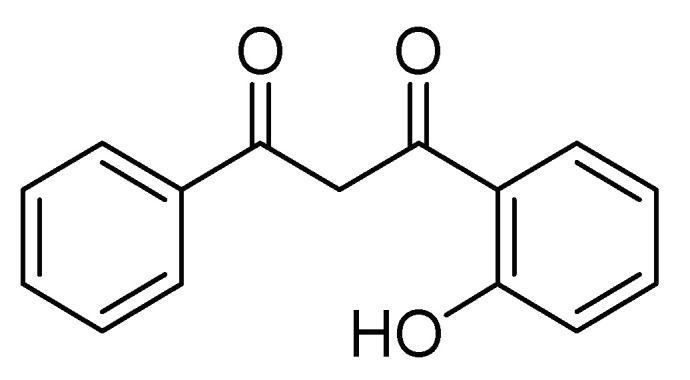
Base structure of synthetic flavonoid derivatives.

Some synthetic derivatives from natural metabolites, such as flavonoid derivatives containing chromone (4*H*-Benzopiran-4-one), are another kind of insecticide. These compounds are part of natural metabolites with a wide distribution in green plants. The flavonoids have a protector effect from ultraviolet light and microbial damage, and some synthetic derivatives have shown insecticide activity. For example, Romanelli et al. [27] obtained a series of 9 flavonoid derivatives of 1-(2-hydroxyphenyl)-3-aryl-1,3-propanediones. Its base structure is shown in Figure 3. These compounds were evaluated in 2000 mg/kg doses on larvae of *S. frugiperda* in the first stage, applying the mixture by aspersion on 1.5-cm maize leaves. Mortality and time of death were evaluated, and the authors concluded that halogenated flavones had insecticide activity.

**Figure 4:**
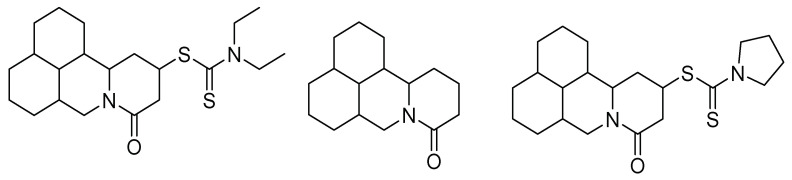
Chemical structure of active molecules derived from matrine.

In this same sense, new compounds have been synthesized from active molecules of natural origin with insecticidal activity, such as matrine, a heterocyclic compound derived from quinolizidine isolated from the roots of *Sophora flavescens* (Aiton) and *Sophora alopecuroides* L. These molecules were introduced to groups of 1-pyrrolidinecarbodithioate and diethylcarbamodithioate to improve their activity through chemical synthesis, enhancing this activity at low concentrations; the structures of these new matrine derivatives are shown in Figure 4 [1].

**Figure 5:**
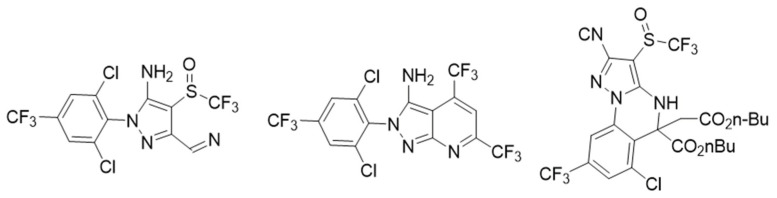
Heterocyclic compounds analogous to fipronil as GABA antagonists.

Other compounds tested are gamma-aminobutyric acid (GABA) antagonists, which resulted in an exciting drug target in lepidopteran and other plagues. Dent et al. [28] discovered the new heterocyclic compounds shown in Figure 5 with high insecticide activity using the “competitive-intelligence-inspired scaffold-hopping” method to obtain fipronil analogs known to be GABA antagonists. These new heterocyclic aryl amines (HAA) showed a broad spectrum of activity on second instar larvae of a set of chewing insect pests. About 370 modifications of the HAA central structure were made, finding a 7-pyrazolopyrimidine lead molecule with better activity against a group of plague insects. Its effectiveness was 2–4 times better in field tests than the commercially available standards. In the search for new insecticides, 4,5-dihydropyrazolo [1,5-*a*] quinazoline derivatives have also been included as GABA receptor antagonist inhibitors, resulting in a mortality of up to 79.63% [29].

**Figure 6:**
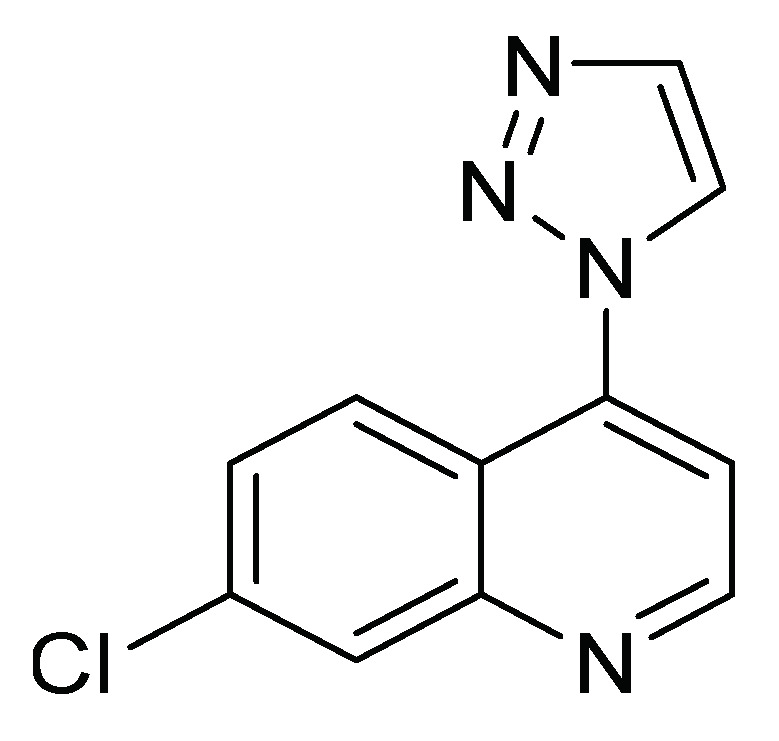
Quinoline derivatives with insecticide activity.

Derivatives of 7-chloro-4-(1*H*-1,2,3-triazol-1-yl) quinoline (Figure 6) have been evaluated on the fourth instar of *S. frugiperda* to explore their insecticidal and antifeedant activity and acetylcholinesterase inhibition. This family of compounds has become a structure of interest for the search and design of new bioactive compounds in medicinal and agricultural chemistry [30]. The characteristics of each of the biological evaluations are listed in Table 1.

## 3. Extracts and Metabolites from Plants

Plants synthesize and release metabolites as a defense mechanism. The produced plant metabolites can be classified into different families according to different chemical groups, such as saponins, tannins, alkaloids, and di- and triterpenoids. These can have an inhibitory effect on many insects, acting as repellent, antifeedant, ovicidal, insecticide, cellular toxicity inducer, mortality inducer, reproductive suppressor, fertility and fecundity reducer, and growth inhibitor. Therefore, many reports indicating the use of extracts of several types of plants against *S. frugiperda* consider these as effective, less expensive, and safer options for the environment and health. Some important metabolites contained in plant extracts and the effective doses found are described below [31,32].

**Figure 7:**
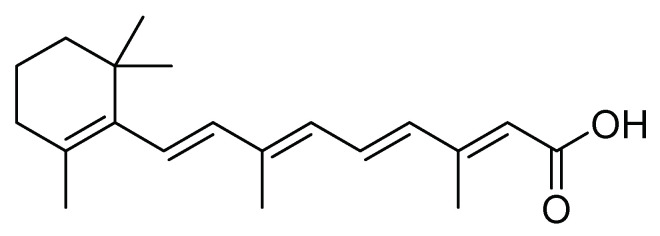
Retinoid: metabolite with an insecticidal and antifeedant effect.

Lizarazo et al. [33] evaluated with the second instar larvae of *S. frugiperda* (corn biotype) the insecticide and antifeedant effect of metabolites present in ethanolic, and dichloromethane extracts obtained from the plants *Polygonum hydropiperoides* L., *Solanum nigrum* L., and *Calliandra pittieri* (Standl.). With the dichloromethane extract of *P. hydropiperoides* at different doses (1 mg/L, 2.5 mg/L, and 5 mg/L), they found that 2.5 mg/L had the best insecticidal and antifeedant effect on larvae of *S. frugiperda* with a mortality rate of 100% 12 days after application, and an antifeedant effect, represented by the consumption of maize foliage below 4%. The authors indicated that one of the metabolites is retinoid (Figure 7), which affects the nervous system and cell respiration in insects [33].

**Figure 8:**
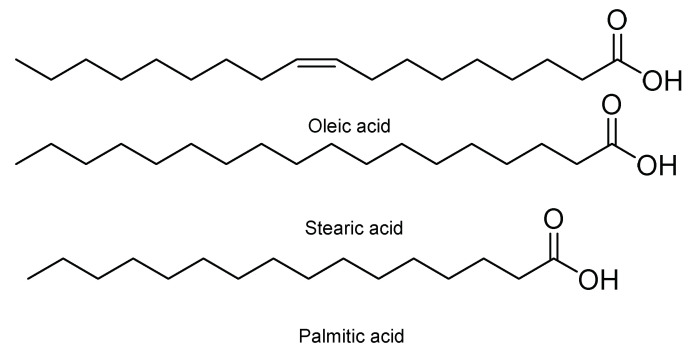
Metabolites in *Carica papaya* varieties Maradol, Mamey, Yellow, and Hawaiian with an antifeedant effect.

Moreover, it has been observed that some seeds of different fruits, such as papaya and orange, have an insecticidal food effect against first instar larvae of *S. frugiperda*. Such is the case of seeds of *Carica papaya* L., varieties Maradol, Mamey, Yellow, and Hawaiian. In powder form and at concentrations of 10 and 15%, they have larvicidal activity [34]. *Carica papaya* var. Maradol extracts in chloroform have also been evaluated. This insecticidal activity has been associated with three metabolites, namely palmitic, oleic, and stearic acid (Figure 8) [35]. Other seeds with antifeedant effects are those of *Citrus sinensis* (L.) Osbeck and *Citrus limonia* (L.) Osbeck. Their metabolites extracted from both seeds and rinds are structurally related polyphenolic compounds and polysaccharides, which have a strong antifeedant and anti-nutritional effect against *S. frugiperda* [36].

**Figure 9:**
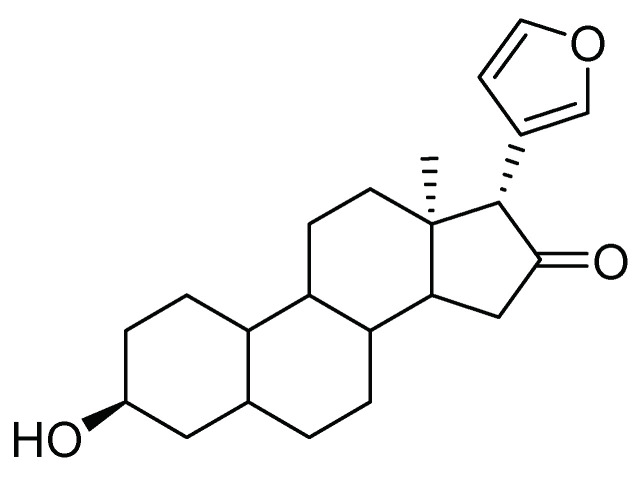
The characteristic structure of limonoids metabolites with antifeedant and toxic effects.

Limonoids are the most representative metabolites of the Rutales order, including the families Rutaceae, Meliaceae, and Simaroubaceae. Their chemical structure is tetra-nortriterpenoides, with a 4,4,8-trimethyl-17-furanyl-steroid backbone, as shown in Figure 9, with several oxygenated functions and a wide variety of biological activities, including anti-fungal, anti-bacterial, and insecticidal. In a report from Argentina, with a plant of the Meliaceae family, the antifeedant and toxic effect of *Melia azedarach* L. extract was evaluated. That extract caught entomological attention because of its excellent properties for biological control attributed to the presence of limonoids that have a known antifeedant effect [37]. Other authors have reported critical antifeedant activity in citrus-derived limonoids, such as *Citrus limon* L. seeds against fifth instar larvae of *S. frugiperda* [38].

**Figure 10:**
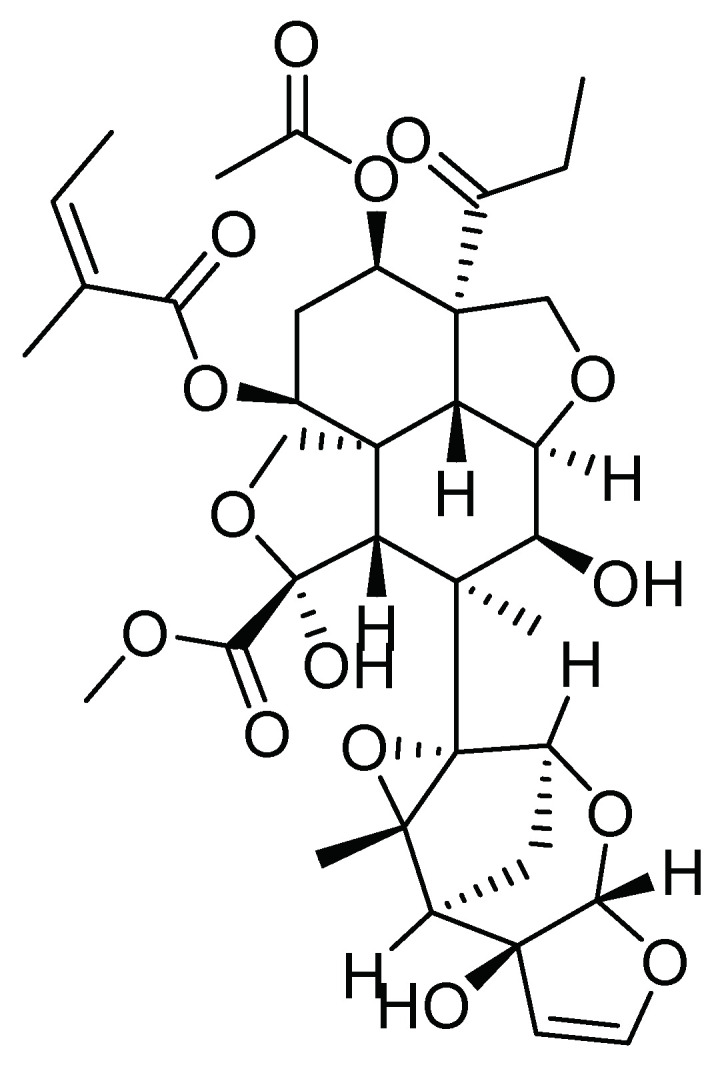
Azadirachtin: a major active metabolite of *Azadirachta indica*.

Metabolites present in *Azadirachta indica* (A. Juss) have been reported with pesticidal capacity against different stages of *S. frugiperda*, presenting antifeedant and repellent activity. The primary chemical constituents of neem are terpenes and limonoids [37]. Considering the above, Trujillo-Ruiz [39] evaluated ethanolic extracts of cellular suspensions from *Azadirachta indica* at different concentrations (2500, 5000, 10,000, and 30,000 ppm), reporting a lethal effect on the second instar larvae of *S. frugiperda*. Furthermore, the ovicidal activity of azadirachtin (Figure 10), a major active metabolite of *Azadirachta indica*, along with a naturally occurring substance called spinosad from *Saccharopolyspora spinosa* (Mertz and Yao, 1990), and methoxyfenozide, a chemical agent, were evaluated at different concentrations on *S. frugiperda* egg masses of less than 48 h of age. The results show that spinosad and azadirachtin had a higher ovicidal effect at a concentration of 1000 mg/L, resulting in mortality in a range between 12 and 31% [19].

**Figure 11:**
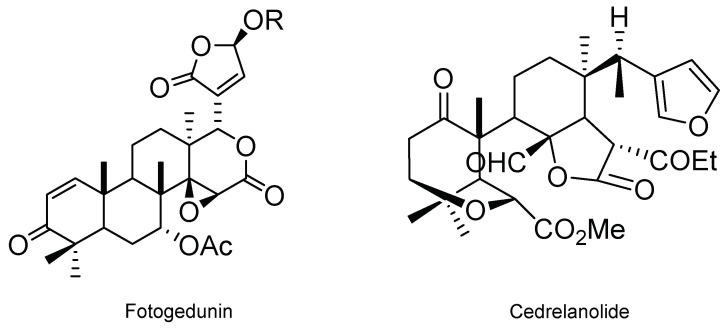
Metabolites extracted from two *Cedrela* species (*Cedrela salvadorensis* and *Cedrela dugessi*).

Azadirachtin belongs to a group of so-called secondary metabolites, limonoids. Therefore, interest in studying these metabolites as an alternative to control *S. frugiperda* has emerged. In this regard, Cespedes et al. [40] isolated an epimeric mixture of fotogedunin, gedunin, and cedrelanolide from two *Cedrela* spp. (*Cedrela salvadorensis* (Standl.) and *Cedrela dugessi* (S. Watson)) for evaluation against first instar larvae of *S. frugiperda*; their structures are shown in Figure 11. The authors determined that gedunin, the epimeric mixture fotogedunin, and a mixture of fotogedunin acetates cause mortality on *S. frugiperda* neonate larvae with LC50 values of 39.0, 10.0, and 8.0 ppm after seven days of exposure, respectively, and cause weight reduction in the pupa, as well as inhibition of larval growth with results comparable to toosendanin, a triterpenoid derivative [40].

**Figure 12:**
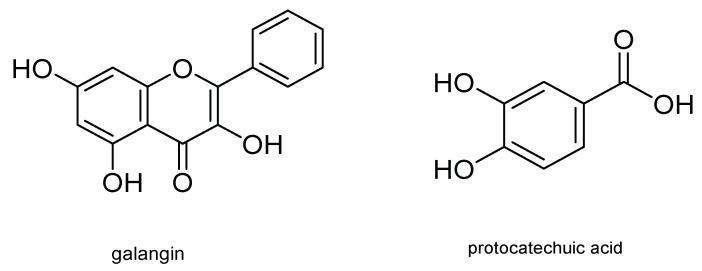
Metabolites present in *Piper subtomentosum* with potential insecticidal activity.

Other metabolites with insecticidal activity are those obtained from the aerial part of the plant of the genus *Piper*. Secondary metabolites from several species of this genus have shown insecticidal activity against Coleoptera, Hymenoptera, and Lepidoptera, including *S. frugiperda*. In this regard, *Piper subtomentosum* (Trel. and Yunck.) metabolites were isolated by bioassay-guided fractionation. These metabolites can disrupt normal biochemical cell processes, producing cell death and therefore death of the *S. frugiperda* larvae stage. Five flavonoids, namely uvangoletin, galangin, chrysin, 5-hydroxy-4,7-dimethoxy-flavones, and pinostrobin; an amide, *N*-*p*-coumaroyl-tyramine; an acylglycerol, monopalmitin; an acid derivative, protocatechuic acid; and a sterol, glycosylated daucosterol, were extracted and evaluated against first instar larvae of *S. frugiperda*. The most active metabolites were galangin and protocatechuic acid. Their structures are shown in Figure 12 [41].

**Figure 13:**
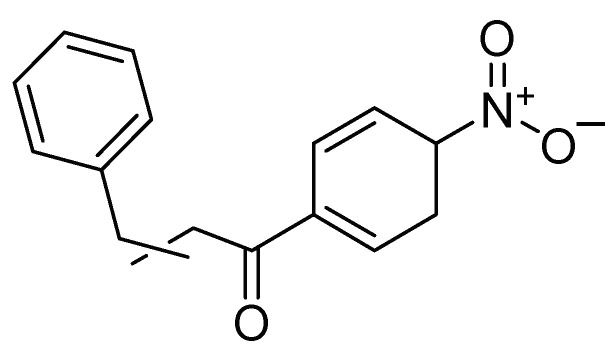
An active compound derived from piperidine present in *Piper piressi*.

Other authors have synthesized and evaluated the toxic effect of 11 amides on second instar larvae of *S. frugiperda*; two were of natural origin, isolated from *Piper piressi* (Yunck.); the most active amide was the natural derivative of piperidine 4 with a DL50 of 1.07 µg/mg on larva [42]. Castral et al. [43] obtained an indexed combinatorial library of amides and evaluated the toxic effect of these compounds on second instar larvae of *S. frugiperda*. (*E*)-1-(1-piperidinyl)-3-[4-(trifluoromethoxy) phenyl-2-propen-1-one was the most active with a DL50 of 0.793 µg/mg. This same amide (Figure 13) was also evaluated by ingestion, and at the lowest concentration (1 mg/kg), produced a mortality of 83.3% [43].

**Figure 14:**
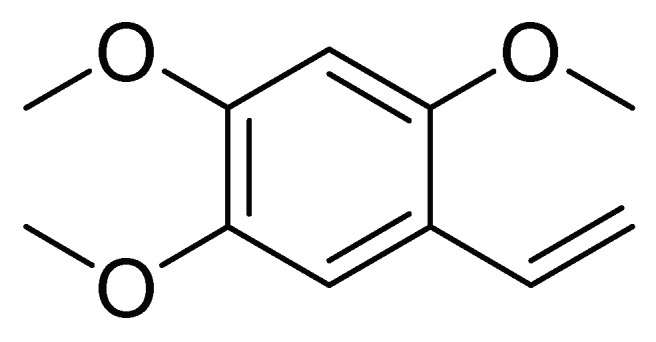
Structure of the active 2,4,5-trimethoxystyrene metabolite from *Duguetia lanceolata*.

In another work, Alves et al. [31] investigated the activity against first instar larvae of *S. frugiperda* of 19 dichloromethane soluble fractions obtained from the metabolic extracts of 10 species of the Annonaceae family. The crust of the stem of *Duguetia lanceolata* (A. St.-Hil). showed higher insecticidal activity with an LT50 of 61.4 h and an LC50 of 946.5 µg/mL. Then, another ten *D. lanceolata* specimens were analyzed by metabolomics and by uni and bidirectional RMN spectroscopy. The results indicate that the effect could be attributed to 2,4,5-trimethoxystyrene (Figure 14), suggesting that this compound may be implicated in the insecticide activity of the crust stem fraction of *D. lanceolata* [31].

The insecticidal activity of common pesticidal plants was recently evaluated on a population of second instar larvae of *S. frugiperda* from Malawi, Africa. Ten extracts of the plants *Azadirachta indica*, *Ocimum basilicum* L., *Nicotiana tabacum* L., *Cymbopogon citratus* (DC.) Stapf., *Tephrosia vogelii* (Hook.f.), *Aloe vera* (L.) Burm.f., *Lantana camara* (L.), *Trichilia emetica* (Forssk.) Vahl, *Vernonia amygdalina* (Delile), and *Lippia javanica* (Burm.f.) Spreng were used. In the contact toxicity tests, the highest larval mortality was obtained from *Nicotiana tabacum* (66%) and *Lippia javanica* (66%) and by ingestion of *L. javanica* (62%) and *N. tabacum* (60%) at a concentration of 10% [44]. The characteristics of the biological evaluations are presented in Table 2.

## 4. Biological Control

Biological control involves using organisms or their components in pest control. This strategy is based on the natural principle that many species feed, live, and reproduce at the expense of others whose populations are regulated by the first who arrive in the different ecosystems [48]; the term biological control is classified according to the mode of action or process involved as conservation biological control and inoculative or inundative biological control [49].

One of the most studied organisms in biological control is the bacterium *Bacillus thuringiensis* (Bt). After its exponential growth phase, this bacterium produces a subapical spore and one or more parasporal bodies and composite inclusions of one or more crystal proteins (ICPs) that have specific insecticidal activity, even at the species level [50]. Transgenic plants have been developed to control *S. frugiperda*, and populations resistant to Cry1 proteins have been characterized in Brazil, Argentina, Puerto Rico, and the southeastern United States [51]. The implementation of Bt in corn and cotton crops with the Cry1A protein has demonstrated an ability to develop strong tolerance quickly. It has been shown that it may not be desirable to use an identically designed biological control for all Lepidoptera species [52].

The susceptibility of *S. frugiperda* to toxins Cry1Ab, Cry2Ab, Cry1Fa, and Vip3Aa has been also studied [53,54]. In recent years, second-generation Bt crops have been introduced. These crops, which combine more than one insecticide protein gene in the same plant, provide better pest control. Some of the new combinations include the expression of the genes Cry and Vip. The Cry and Vip proteins have different targets in the insect gut and possibly different toxicity mechanisms [55,56]. Other work on the synergism between the Cry1Ab, Cry1Fa, and cadherin (SfCad) proteins has been done using CRISPR/Cas 9 genome editing technology and Bt toxin cytotoxicity assays in an insect cell line. It has been suggested that cadherin (SfCad) of *S. frugiperda* is not involved in the mode of action of toxins Cry1Ab and Cry1Fa [57].

Another bacterium used for biological control against *S. frugiperda* is *Saccharopolyspora spinosa*, class Actinobacteria, which by aerobic fermentation produces spinosyn A and D. This active ingredient acts on the nervous system of insects, causing high activity and excitement, including involuntary muscle twitching, tremors, prostration, fatigue, and death after 72 h. Thus, it has established itself as a control strategy against *S. frugiperda* and other nematode pests of vegetables [58,59].

Entomopathogenic viruses have also been used as biological controls. These emerged as promising and environmentally sustainable alternatives due to their high specificity and virulence. For this strategy, viruses need to be ingested by the insect to cause illness and subsequent death. Recombinant baculoviruses have become an efficient vector, which could be used to produce a protein of interest in insect cell cultures [60]. The symptoms are loss of appetite, lethargy, body sagging, softening of the integument, and a tendency to turn off color. Some viruses studied against *S. frugiperda* are Rhabdovirus (Sf-RV) [61]; granulovirus (SfGV ARG) type 1, 2, and 3 [62,63]; ascovirus (SfAV-1a) [64]; ichnovirus (HdIV) [65]; and the most studied, nucleopolyhedrovirus (SfMNPV) [55,66]. Undoubtedly, this strategy has shown high efficiency in the control of *S. frugiperda* in in vitro evaluations. However, one of the limitations of implementing viruses is their instability when applied in the field due to their susceptibility to ultraviolet radiation and other environmental factors such as temperature, humidity, and pH [67].

The fungus that stands out because of its entomopathogenic power is the ascomycete *Beauveria bassiana* (Bals.-Criv.) Vuill., responsible for producing the disease white muscardine. This fungus is parasitic by adherence to the insect cuticle, produces conidia that germinate, and creates a hyphae network inside the insect. These have specific reactions that cause the death of the host insect [68]. In this regard, the toxicity of Micoralis^®^, a commercial bioinsecticide made from *B. bassiana* (miscible liquid, *B. bassiana*, 2.3 × 10^7^ spores/mL in 1.67% of the product) was evaluated against first instar larvae of *S. frugiperda*. The results showed low mortality at the concentrations tested with the highest mortality, 48%, occurring after 120 h (6 days) at a concentration of 1 × 10^9^ spores/mL, with an IC_50_ value of 1.3 × 10^8^ spores/mL [69]. Within the kingdom Fungi, we also find the entomopathogenic fungus *Metarhizium anisopliae* (Metschn.) Sorokin, a deuteromycota filamentous green olive fungus that causes green muscardine disease, which damages a wide variety of insects (300 species of Coleoptera order, lepidoptera, and Homoptera), *Metarhizium anisopliae* [70].

More recently, synergistic mortality and fungal performance between chemical and biological control have been evaluated. The combination of low doses of insecticides and entomopathogenic fungi can improve integrated pest management programs [71]. Another fungus of interest with biocontrol activity is *Nomuraea rileyi* (Farl.) Samson; however, despite its high activity, up to 90% control in *S. frugiperda* larvae, it has not been commercialized as any formulation [72]. This organism acts by contact, invading the insect’s body and causing death, making it a promising alternative to control *S. frugiperda* larvae. Table 3 shows the information on the studies carried out.

Other organisms that participate in biological control are natural enemies, such as parasitoids, which have proven their importance as population regulators of *S. frugiperda* [75,76]. *Spodoptera frugiperda* have more than 100 species of parasitoids. In Mexico, more than 88 have been registered [77]. The species most frequently detected are *Trichogramma* spp., *Chelonus* spp., *Apanteles* spp., *Cotesia marginiventris* (Cresson, 1865) (Hymenoptera: Braconidae), *Meteorus laphygmae* (Viereck, 1913) (Hymenoptera: Braconidae), *Euplectrus* spp., *Ophion* spp., *Campoletis* spp., and several species of parasitic flies, as well as the families Sarcophagidae and Tachinidae, among which *Archytas marmoratus* (Townsend, 1915) (Diptera: Tachinidae) and *Lespesia archippivora* (Riley, 1871) (Diptera: Tachinidae) are found. Other parasitoids reported against *S. frugiperda* are some species of the genera *Trichogramma*, *Spalangia*, *Cochliomyia*, *Ceratitis*, and *Nomuraea*. In Colombia, it was found that *Coleomegilla maculata* (De Geer, 1775) (Coleoptera: Coccinellidae) was the most common predatory species of this pest insect [78]. Other reported predators are *Zelus* sp., *Orius* sp., *Podisus* sp., *Chrysoperla* sp., *Dorus taeniatum* (Dermaptera: Forficulidae)*, Trichogramma atopovirilia* (Oatman and Platner, 1983) (Hymenoptera: Trichogrammatidae), and *Trichogramma pretiosum* (Riley, 1879) (Hymenoptera: Trichogrammatidae) [77].

In the case of the parasitoid *Chelonus insularis* (Cresson, 1865) (Hymenoptera: Braconidae) in Mexico, it is the most notable species, exercising parasitism of 86% in some regions of the State of Morelos. In conjunction with *Chelonus* sp., they are the most prevalent in North America. A more diverse family is Ichneumonidae, followed by Braconidae [79] in Chiapas, Mexico, finding a trend of 31.8%. Prevalent parasitoid species with a value of 77% were *Chelonus insularis* with 344 emerging parasitoids. Other species were *Eiphosoma vitticolle* (Cresson, 1865) (Hymenoptera: Ichneumonidae) (11.0%), *Euplectrus plathypenae* (Howard, 1885) (Hymenoptera: Eulophidae) (7.6%), and *Ophion flavidus* (Brulle, 1846) (Hymenoptera: Ichneumonidae) (2.6%), followed by a few individuals, namely *Pristomerus spinator* (Fabricius, 1804) (Hymenoptera: Ichneumonidae), *Meteorus* sp., and the tachinid, *Lespesia archippivora*. In other states of Mexico, *Meteorus laphygmae* and *Pristomerus spinator* have been reported as parasitoid larvae with higher rates of parasitism in populations in Sinaloa (22.2%) and Michoacan (22.1%). *P. spinator* has also been found in Altamira, Tamaulipas with 10% parasitism [76].

In a study in the United States, 8353 *S. frugiperda* larvae were collected from 3 south Florida counties to identify the most common parasitoids. *Cotesia marginiventris* and *Chelonus insularis* were the most detected with 23 and 18 of the 25 sample sites, respectively. Other parasitoid species detected were *Aleiodes laphygmae* (Viereck, 1912) (Hymenoptera: Braconidae), *Euplectrus platyhypenae*, *Meteorus* spp., *Ophilon flavidus* (Brulle, 1846) (Hymenoptera: Ichneumonidae), and nonidentified species of Tachinidae. Parasitism was comparable between summer and autumn, but it was most prevalent in nontreated fields (44.0 ± 9.6%) than in fields treated with insecticides (15.0 ± 2.5%) [80].

Larval parasitoids such as *Coccygidium luteum* (Saussure, 1892) (Hymenoptera: Braconidae) and *Drino quadrizonula* (Thomson, 1869) (Diptera: Tachinidae) have been recorded in South Africa with a maximum parasitism of 23.68% and 8.86% [81]. In addition, in South Africa, Ivory Coast, Niger, Benin, and Kenya, *Telenomus remus* (Nixon, 1937) (Hymenoptera: Scelionidae) has been reported as an essential parasitoid of fall armyworm eggs [82]. Some others reported in these regions are *Trichogramma* sp., *Chelonus bifoveolatus* (Szepligeti, 1914) (Hymenoptera: Braconidae), *Coccygidium luteum*, *Cotesia icipe* (Fernandez-Triana and Fiaboe, 2017) (Hymenoptera: Braconidae), *Meteoridea testacea* (Granger, 1949) (Hymenoptera: Braconidae), *Charops* sp., *Metopius discolor* (Tosquinet, 1896) (Hymenoptera: Ichneumonidae), *Pristomerus pallidus* (Thomson, 1890) (Hymenoptera: Ichneumonidae), and *Drino quadrizonula* [83].

Parasitoids such as *Campoletis grioti* (Blanchard, 1946) (Hymenoptera: Ichneumonidae)*, Chelonus insularis*, and *Archytas marmoratus* have been reported in localities of Argentina as frequent parasitoids, and some others, such as *Archytas incertus, Ophion sp*., *Euplectrus platyhypenae*, and *Incamyia chilensis.* In this study, the authors analyzed the variations between parasitoids in different localities over five years. They reported that the diversity of parasitoids could be attributed to various factors such as insecticides, agricultural and cultural practices, natural enemies, alternative hosts, and climatic factors [84].

**Figure 15:**
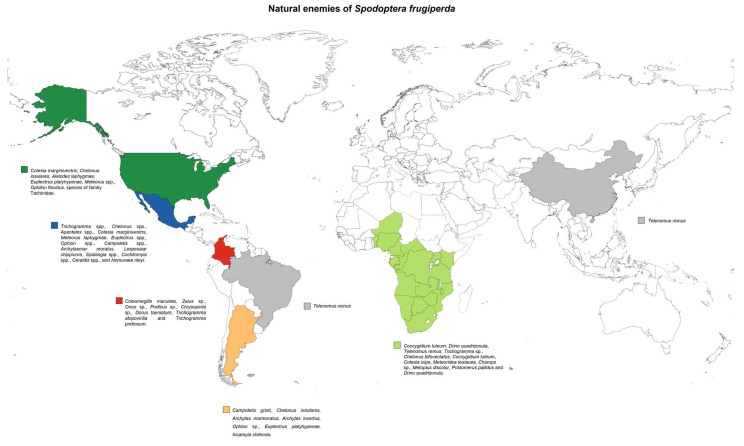
Geographical distribution of the leading natural enemies reported or used in the control of *S. frugiperda* worldwide.

In late 2019, *S. frugiperda* established itself in southern China and now persists throughout the year. In China, as in Brazil, one of the natural enemies being implemented is *Telenomus remus* [85]. The distribution of the natural enemies commonly found or used to control *S. frugiperda* in the countries where this pest insect has currently appeared is shown in Figure 15.

These natural enemies of *S. frugiperda*, which can parasitize eggs, egg and larvae, and only larvae, are candidates for selection in an augmentative biological control program; based on their reproductive performance, host selection, resilience at low host population densities and dispersal capabilities, they can be selected to be mass reproduced in a laboratory and preserved in strips with a high density (>2500 eggs/in^2^) of parasitized eggs of alternative hosts and be released in strategic areas (20 to 40 points per hectare) to allow the search for eggs or larvae of *S. frugiperda* and to carry out their control. Its low cost and the fact that the evidence suggests that these parasitoids do not show a preference for the rice or corn biotype make it a viable option for the control of *S. frugiperda*, in addition to that fact that they may be a viable option in areas of new invasion such as Africa, where it has no associated natural enemies [86,87,88].

## 5. Monitoring Methods

Monitoring refers to tracking the presence, density, distribution, and severity level of the infestation caused by pest insect in a certain ecosystem, with the objective of carrying out an intervention safe to protect crops, preserving ecological balance, and minimizing damage to the environment [86]. The monitoring of *S. frugiperda* can be carried out through light traps, pheromones traps, and regular scouting.

Light traps take advantage of the fact that *S. frugiperda* is attracted to light sources, allowing their monitoring and control [89]; although it has been shown that the capture rate of males and females of *S. frugiperda* is lower than that of other lepidopterans, due to its phototactic behavior [90], recently this strategy has been combined with other control methods, such as “Push–pull” (cultural method, which protects crops by intercropping them with pest-repellent plant species), capturing a greater number of *S. frugiperda* individuals than only using light traps, deterring their entry into the crop fields [91].

The sex pheromone traps use chemical signals that travel by the air great distances to attract male insects, allowing for their monitoring [92]. To date, in glands of *S. frugiperda* females from different regions, it has been possible to identify active components of sex pheromones such as (Z)-9-tetradecenyl acetate (Z9-14:OAc) as a major component, (Z)-7-dodecenyl acetate (Z7-12:OAc), (Z)-11-hexadecenyl acetate (Z11-16:OAc), (Z)-9-dodecenyl acetate (Z9-12:OAc), and (E)-7-dodecenyl acetate (E7-12:OAc), the latter a characteristic active component in Brazilian populations [93,94]; although the variations in the presence of these components is low, various authors have described differences in the composition of sex pheromones of *S. frugiperda* depending on the geographical area, recommending the optimization and formulation of lures with specific pheromonal components for correct monitoring [95]. Monitoring based on pheromone traps has been shown to be effective to predict where and when an infestation of *S. frugiperda* might develop, detecting male populations as a sign of the subsequent appearance of eggs and larvae, allowing farmers to decide the time and number of pesticide applications, avoiding unnecessary actions [96]. Cruz et al. [97] demonstrated that the use of monitoring based on pheromone traps as a decision-making tool to the control of *S. frugiperda* in a corn crop is feasible, since they obtained up to 91% of larvae mortality when applying chemical insecticides due to the early capture of at least three males with the insect traps and not until the observation of damage in crop, 10–20% of pinhole-type and shot hole-type damage; the monitoring with pheromone traps allowed the application of the chemical insecticide in the time when the larvae were between the third and fourth instar, susceptible stages, unlike the application based in damage observation (43 day after) in which the larvae already were more resistant and could avoid this control method. 

The regular scouting commonly performed by a farmer implies the field inspection through protocols based on the growth stage of the crop, looking for egg-hatch and leaf damage caused by larvae of *S. frugiperda*, with the objective of obtain the percentage (%) of infested plants and to determine accordingly action thresholds if necessary to apply control measures; for example, in maize, if 20% of the seedlings are infested, the application of chemical insecticide is justified [86]. The latest studies on this topic focus on examining and testing the efficiency of new scouting patterns to the traditionally used “W”, “Ladder”, and “Diagonals”; however, no significant differences have been identified in the sampling with the new proposals [98].

The success of the control methods addressed in this review can be increased if they are combined with previously established monitoring methods in a given geographic area. This is because, for example, monitoring in combination with an inundative control method through the release of parasitoids would allow one to synchronize their release with the target stage of the insect (egg, larva), and carry out the biorational application of pesticides, botanical insecticides, or biopesticides when the density population of *S. frugiperda* could cause major economic damage [86].

## 6. Conclusions

The strategies studied for the control of *S. frugiperda* are diverse (chemical and natural products and biological controls). However, the adaptability and resistance of the insect have hindered the success and favored the geographical spread of *Spodoptera frugiperda*. Chemical control can hardly be substituted; however, optimization strategies to find new chemical compounds with substituents that help lower toxicity and maintain insecticidal activity at low concentrations are still necessary. In this regard, one of the promising chemical families is fipronil analogs, which act as GABA antagonists and reflect the timely search for inhibitors of specific enzymes of the pest insect.

On the other hand, natural products show biological activity at higher concentrations than chemical products. However, they have provided the guideline to identify active secondary metabolites to obtain new synthetic or semisynthetic chemicals with more significant biological potential for the control of *S. frugiperda*, as in derivatives of synthetic amides and limonoids from organic extracts.

Biological control using organisms, or their components, has achieved an efficient strategy for managing *S. frugiperda*, such as the creation of Bt crops. However, the search and improvement of these have become continuous work, such as the search for insecticidal proteins to create second-generation Bt cultures that combine more than one protein gene in the same plant. In this same sense, the pathogenicity mechanisms of other mentioned microorganisms are analyzed. Additionally, parasitoids play a vital role in the integrated management system of this pest in different parts of the world, finding that in the region of the United States and the northern region of Mexico, the presence of the genus *Chelonus* predominates, and in Central and South America, the presence of *Trichogramma sp.* We can see that in non-endemic regions where *S. frugiperda* is currently distributed, the use of *Telenomus remus* predominates, as in Brazil.

Based on the above, we emphasize that it is necessary to continue with the improvement, design, and development of new strategies to control *S. frugiperda* in every one of the mentioned items. These strategies will allow us to have more effective options against the pest insect, and its combination with the monitoring methods could increase success. However, above all, it will enable us to devise new opportunities that are more environmentally friendly.

## Figures and Tables

**Table 1:** Chemical agents with potential insecticidal activity against *S. frugiperda*.

Control Agents	Application Method	Dose	Activity	Reference	Country
Matrine and derivatives	In vivo	0.648 mmol/L 1.13 mmol/L	Apoptosis induction	[1]	China
Emamectin benzoate	Ingestion	0.025 mg/L	Acetylcholinesterase inhibitors	[16]	China
*N*′-tert-butyl-*N*′-(3,5-dimethylbenzoyl)-3-methoxy-2-methyl benzohydrazide	Ingestion	500 mL/ha	Insecticide Induce premature molting and cause death	[18]	Brazil
*N*-oxide benzofuroxan methyl-5-carboxylate *N*-oxide derivatives	Ingestion	0.328 mg/mL 0.229 mg/mL 0.289 mg/mL	Insecticide esterase inhibitor	[23]	Mexico
Flavone derivative of 1-(2-hydroxyphenyl)-3-aryl-1,3-propanedione	Topical	200 mg/kg	Insecticide Modulation of feeding and oviposition of the insect	[27]	Argentina
Aril amine heterocyclic-7-pyrazolo pyridine	Ingestion	0.85 µg/cm^2^	Insecticide GABA antagonists	[28]	United States of America
5-acetyl-8-chloro-5-(3-hydroxypropyl) -7-(trifluoromethyl)-3-((trifluoromethyl)sulfinyl)-4,5-dihydropyrazolo [1,5] quinazoline-2-carbonitrile	Ingestion	100 mg/L	Insecticide antagonists GABA	[29]	China
4-(4-methyl phenyl)-1*H*-1,2,3-triazolyl-quinoline	Ingestion	0.65 mg/g insect	Acetylcholinesterase inhibitors	[30]	Colombia

**Table 2:** Metabolites and extracts from plants with potential insecticidal activity against *S. frugiperda*.

Control Agents	Application Method	Effective Dose	Effectivity/Stage	Reference	Country
*Azadirachta indica* extract	Ingestion Topical	14.79 mg i.a. kg^−1^ diet 7.06 µg i.a. g^−1^ larvae	Ovicidal	[19]	Mexico
*Duguetia lanceolata* extract	Ingestion	946.5 µg/mL	Insecticidal	[31]	Brazil
*Polygonum hydropiperoides* extract	Ingestion	2.5 mg/L	Insecticidal and antifeedant	[33]	Colombia
*Carica papaya* extract	Ingestion	10–15%	Larvicidal	[34]	Mexico
*Citrus sinensis* and *C. limonia* extract	Ingestion	0.75–1.0%	Antifeedant and antinutritional	[36]	Colombia
*Citrus limon* limonoids (limonina and obacunona)	Ingestion	0.05 M	Antifeedant	[38]	Italy
*Citrus limon* limonoids (limonol, liomonin, 7-oxime limonin, and methoxime)	Ingestion	0.05 M	Antifeedant	[38]	Italy
*Azadirachta indica* extract	Ingestion	2.256 ppm 3.928 ppm 2.818 ppm 1.064 ppm	Antifeedant and repellent	[39]	Colombia
*Cedrela salvadorensis* and *C. dugessi* metabolites (fotogedunin, gedunin, and cedrelanolide)		39.0 ppm 10.0 ppm 8.0 ppm	Insecticidal	[40]	Mexico
*Piper piressi* amide (*N*-[3-(3′,4′-methylenedioxyphenyl)-2-€-propenoyl] piperidine)	Ingestion	1.07 µg/mg larvae	Insecticidal	[42]	Brazil
Natural and synthetic amides of Piper (E)-1-(1-Piperidinyl)-3-[4-(trifluoro methoxy)phenyl]-2-propen-1-one)	Ingestion	0.793 µg/mg larvae	Insecticidal	[43]	Brazil
*Lippia javanica, Nicotiana tabacum*	Ingestion Contact	10%	Insecticidal	[44]	Africa
*Melia azedarach* extract	Ingestion	2000 µg/cm^2^	Antifeedant	[45]	Argentina
*Piper cenacladum* amides (piplartine, 4′-desmethylpiplartine)	Ingestion	Piplartine: 0.203 g 4′-desmethyl piplartine: 0.1575 g	Antifeedant	[46]	United States of America
*Piper tuberculatum* extract	Ingestion	219 mg/insect	Insecticidal	[47]	Brazil

**Table 3:** Microbial agents with potential insecticidal activity against *S. frugiperda*.

Control Agents	Application Method	Effective Dose	Effectivity/Stage	Reference	Country
Protein *Cry* and *Vip B. thuringiensis* Proportion Vip3Aa:Cry1Ca 1:0 0:1 1:2	Ingestion	0.44 µg/cm^2^ 0.052 µg/cm^2^ 0.30 µg/cm^2^	Insecticidal	[55]	Brazil
*Saccharopolyspora spinosa*	Ingestion	0.3 and 1.0 g IA/ha	Insecticidal	[59]	Mexico
Granulovirus SfGV (VG008)	Ingestion	4.5 × 10^5^ OB/mL for 29 days	Insecticidal	[62]	Colombia
Granulovirus	Ingestion	1.0 × 10^8^ OB/mL for 14 days	Insecticidal	[63]	Argentina
Ascovirus 1a (SfAV-1a),	Topical	1 × 10^8^/mL for 7 days	Insecticidal	[64]	United States
Ichnovirus (HdIV)	Topical	4 × 10^5^	Insecticidal	[65]	France
*Bauveria bassiana*	Topical	1.3 × 10^8^ spores/mL	Insecticidal	[69]	Mexico
*Metarhizium anisopliae*	Topical	1×10^12^ conidia/ha	Insecticidal	[70]	Mexico
Nucleopolyhedrovirus NVP009 NVP011	Ingestion	2.2×10^5^ CI/mL 7.0×10^5^ CI/mL	Insecticidal	[73]	Colombia
*Nomuraea rileyi*	Ingestion	1.0× 10^7^ conidia/mL	Insecticidal	[74]	Colombia

## Data Availability

Not applicable.

## Sample Availability

Samples of the compounds are not available from the authors.
